# Do New Firms Recruit Employees From Small or Large Firms, and Do Small or Large Firms Recruit Employees From Firms That Cease to Operate?

**DOI:** 10.3389/fsoc.2022.853689

**Published:** 2022-05-13

**Authors:** Jarle Aarstad, Olav Andreas Kvitastein

**Affiliations:** The Mohn Centre for Innovation and Regional Development, Western Norway University of Applied Sciences, Bergen, Norway

**Keywords:** new firm formation, firm closure, firm size inequality, recruitment, panel data, dynamic panel regression

## Abstract

Panel data of Norwegian industries show that when they increase in the number of firms, firm size inequality in employees decreases. Decreasing firm size inequality implies that large firms become smaller in employees, and an increasing number of firms in an industry implies that more new firms are established than closed, i.e., ceasing to operate and going out of business. Thus, new firms chiefly recruit employees from large firms. Similarly, the data show that when industries decrease in the number of firms, firm size inequality in employees increases. Increasing firm size inequality implies that large firms become larger in employees, and a decreasing number of firms in an industry implies that more firms are closed than established. Thus, large firms chiefly recruit employees from firms that cease to operate. An implication of our findings is that large firms are crucial in recruiting employees to new firms and in recruiting employees from firms that cease to operate.

## Introduction

A study by Kacperczyk and Marx (2016) has shown that employees in small firms have a stronger proclivity to establish new firms than employees in large firms. However, the findings are the opposite for firms that cease to operate, i.e., going out of business, as employees in large firms have a stronger proclivity to establish new firms than employees in small firms. A possible explanation of the mixed findings is that large firms hosting abundant entrepreneurial talent (Lee, 2021), i.e., people with a natural aptitude or skill (Cresswell, 2021), have strong resources to keep them, but this possibility vanishes when ceasing to operate (Kacperczyk and Marx, 2016). Consequently, one can infer that large firms, in a superior way, recruit and “mold” entrepreneurial talent while constraining employees to become entrepreneurs unless they cease to operate.

Drawing upon the above arguments, we study in this paper whether new firms, in most cases being inherently small, tend to recruit employees from other small or large firms. Hence, we do not explicitly study whether employees become entrepreneurs but the extent to which they are recruited from small or large firms into new firms. Overall, we assume that new firms chiefly recruit employees from large firms, and the reasons are threefold. First, as large firms likely host “molded” entrepreneurial talent (Kacperczyk and Marx, 2016; Lee, 2021), their employees may be considered particularly attractive to new firms, and, accordingly, there is a likely “pull” effect. Second, we assume a likely “push” effect in recruiting employees from large to new firms. Although recruitment from large to new firms is not necessarily a result of large firms ceasing to operate (hence, operating large firms likely have relatively superior incentives to keep talented employees), new firms are de facto also operating and accordingly represent a relatively low risk for employment (as compared to establishing a new firm from scratch and becoming a de facto entrepreneur). Therefore, the threshold for an employee to transfer from a large to a new firm is relatively low. A third reason the threshold of transferring from a large to a new firm is relatively low might be an assumption of renewed employment opportunity at the previous large firm if the new firm were to cease operating.

Also, we study whether small or large firms tend to recruit employees from firms that cease to operate, i.e., going out of business. Having assumed in the paragraph above that new firms chiefly recruit employees from large firms, we further assume that large firms will reciprocally tend to recruit employees from firms that cease to operate. The reason, we believe, is that because relatively many new firms remain small and cease to operate within a few years of operation (Aldrich, 1999; Wiklund et al., 2010), the “molded” employees they recruited from large firms (cf. our arguing in the paragraph above) will be recruited back to large firms as they likely offer better conditions than small firms (Kacperczyk and Marx, 2016). In addition, we assume that the “molded” employees may have achieved an even higher attractiveness due to further development of their human capital when employed in a new firm. Overall, more small firms cease to operate than large firms (Aldrich, 1999), and large firms may finally be inclined to recruit employees from those numerous small firms that cease to operate independently of whether they were new with “molded” employees from large firms or not. The reason is that employees from small firms generally have high and diverse skills and limited employment opportunities if their current employer ceases to operate. As such, large firms, in general, are in a relatively favorable negotiation position to recruit talented employees when small firms cease to operate. Taken together, we assume that large firms tend to recruit employees from firms that cease to operate. Our assumption partly challenges the results of a survey of managerial perceptions by Kacperczyk and Younkin (2021, p. 1), showing “that [a history of] founding [a new firm] significantly reduces the likelihood that an employer interviews a male [but not female] candidate”. However, a classical study has revealed strong discrepancies in perceptions of people and actual behavior toward them (LaPierre, 1934), and as we study actual employment, we do not rule out that our findings may point to a conclusion, not in line with the study by Kacperczyk and Younkin.

To study our research questions, we do panel data analyses of Norwegian industries between 2001 and 2014. When an industry increases in the number of firms from one year to another, ceteris paribus, more new firms are established than ceasing to operate, and when an industry decreases in the number of firms, ceteris paribus, more firms cease to operate than new ones being established. Thus, increasing and decreasing industry size in the number of firms is our independent variable as it captures net growth in new firm establishments and a net decline in operating firms, respectively. For a firm to be included as an “established” entity in an industry a given year, it needs to report positive operating revenues and positive or negative operating profits. In other words, we do not include firms in our analyses that do not show any sign of substantial economic activity.

To study the recruitment of employees to and from firms of different sizes as a dependent variable, we use the Gini (1936) coefficient of industries' firm size distribution in terms of full-time employees. When an industry decreases in firm size inequality from one year to another, ceteris paribus large firms become smaller in terms of employment, and when an industry increases in firm size inequality, ceteris paribus large firms become even larger.

We argued above in the second paragraph that new firms will chiefly recruit employees from large firms. If this is the case, our industry panel data will show that the firm size inequality will decrease as a function of net growth in new firm establishments. The reason is that as employees in new firms are chiefly recruited from large firms, these large firms' size will shrink in size and therefore reduce the industry's firm size inequality Gini coefficient. Also, we argued above in the third paragraph that large firms will tend to recruit employees from firms that cease to operate. If this is the case, our industry panel data will show that firm size inequality will increase as a function of a net decline in operating firms. The reason is that as employees in firms that cease to operate are chiefly recruited to large firms, these large firms' firm size will increase and therefore increase the industry's firm size inequality Gini coefficient.

We cannot rule out that industries increase and decrease in the number of firms due to demergers, and mergers and acquisitions (M&As), respectively. However, if this is the case, the association between the independent and dependent variables will largely occur coincidingly without a time lag, as employees in the short run will tend to be transferred to the new legal entity. Hence, a time lag between the independent and dependent variables will rule out this alternative explanation. Below, we run our analyses with and without a time lag between the independent and dependent variables to address this issue.

## Materials and Methods

Data for this study was provided by Statistics Norway and the Brønnøysund Register Centre, a public enterprise operating many of Norway's most important registers. We linked employee-level data with data at an enterprise- and industry level. To identify different industries, we used firms' digit-two NACE codes, which is the standard European nomenclature of productive economic activities. The panel includes industries of firms reporting on employment, wages, and operating revenues. Further details concerning the identification of different industries are provided by Aarstad and Kvitastein (2021a).

To calculate the Gini coefficient of industries' firm size distribution at year *t* (*t* = 2001–2014) as a dependent variable, we used each firm's size in full-time employees. Theoretically, assuming that all firms in an industry have the same number of employees, the Gini coefficient is zero, and it takes a value close to one if an industry has one very large firm accompanied by many small ones.

The independent variable is each industry's number of firms at year *t* divided by the average number of firms in each industry for all years included in the aggregated panel. We control for industry employment by including the number of its full-time employees at year *t* also divided by the average number of full-time employees in each industry for all years included in the aggregated panel. Our motive for the divisions is to account for industries inherently different in size concerning the number of firms and employees (Aarstad and Kvitastein, 2021b). As such, we model the relative number of firms and full-time employees as independent and control variables, respectively.

## Results

Reporting fixed effects panel regressions with robust standard errors, Table 1 shows that an increase in an industry's number of firms from year *t*_−1_ to *t* decreases the firm size inequality at year *t* (Model 1). The effect is slightly stronger when the increasing independent variable is lagged by one year (Model 2). Similarly, we observe that a decrease in an industry's number of firms from year *t*_−1_ to *t* increases the firm size inequality at year *t* (Model 3). The effect is stronger when the decreasing independent variable is lagged by one year (Model 4). So far, we can conclude that industries' firm size inequality is a function of both an increase and a decrease in the number of firms, according to our arguments above. In Model 5, we include the independent variable at both year *t* and *t*_−1_ and observe that only the effect at *t*_−1_ is significant. Model 6 shows that the effect of the independent variable at *t*_−1_ is about the same when omitting the independent variable at year *t*. Therefore, we also conclude that there is a time lag between the independent and dependent variables, and it implies that we can largely rule out demergers and M&As as alternative explanations of the empirical findings (cf. our arguing above in the last paragraph of the Introduction). The number of employees positively affects the dependent variable in some models, and in line with these findings, Aarstad and Kvitastein (2021c) show that industries' firm size inequality decreases as employment decreases, but not vice versa.

**Table 1:** Fixed effects panel regressions with robust standard errors and firm size inequality at *t* as dependent variable.

	**Model 1**	**Model 2**	**Model 3**	**Model 4**	**Model 5**	**Model 6**
Number of firms at *t*	−0.074^***^		−0.030^*^		−0.006	
	(0.020)		(0.012)		(0.030)	
Number of firms at *t*_−1_		−0.075^***^		−0.069^***^	−0.069^**^	−0.074^***^
		(0.021)		(0.011)	(0.024)	(0.013)
Number of employees at *t*	0.057^*^	0.051^†^	0.064	0.081^**^	0.046^†^	0.045^*^
	(0.028)	(0.026)	(0.043)	(0.030)	(0.025)	(0.023)
Number of employees at *t*_−1_	0.036	0.033	0.016	0.019	0.045^†^	0.042^*^
	(0.024)	(0.024)	(0.037)	(0.031)	(0.021)	(0.021)
Number of firms at *t* > *t*_−1_	√					
Number of firms at *t*_−1_ > *t*_−2_		√				
Number of firms at *t*<*t*_−1_			√			
Number of firms at *t*_−1_<*t*_−2_				√		
Year dummies included (but not reported)	√	√	√	√	√	√
N industry-year obs./industries	574/67	540/64	203/62	238/67	783/67	783/67
Min./avg./max. obs. per group	1/8.6/13	1/8.4/12	1/3.3/9	1/3.6/9	1/11.7/13	1/11.7/13
F-value	6.35^***^	6.09^***^	29.5^***^	22.9^***^	14.1^***^	10.7^***^
R-sq. within/between	0.347/0.078	0.353/0.017	0.570/0.028	0.535/0.002	0.393/0.113	0.393/0.107

† *p < 0.10*,

* *p < 0.05*,

** *p < 0.01*,

*** *p < 0.001*.

*Conservative two-tailed tests for regressors and robust standard errors in parentheses*.

The findings in Table 1 concerning the association between industries' number of firms and firm size inequality cannot rule out eventual omitted variable bias and reverse causality. To account for these issues, Table 2 carries out dynamic unconditional quasi-maximum likelihood fixed-effects panel models with robust standard errors (for further details, see Kripfganz, 2016; Leszczensky and Wolbring, 2019; Williams et al., 2019). Model 1 in Table 2 confirms that firm size inequality is a negative function of the number of firms in an industry. The less marked effect, however, may indicate omitted variable bias in Table 1, but the inclusion of a lagged dependent variable can also, in general, depress the effect of an independent variable (Achen, 2000). Model 2 in Table 2 shows that firm size inequality, i.e., large firms becoming larger or smaller, does not reversely cause an industry's number of firms. Employment has a positive effect on the number of firms at *t*, and a negative effect at *t*_−1_. According to Kennedy (2005, p. 82), it may indicate a positive short-term and negative long-term effect, but we do not rule out alternative explanations such as multicollinearity. The issue, nonetheless, merits further attention in future research.

**Table 2:** Dynamic unconditional quasi-maximum likelihood fixed effects panel model with robust standard errors.

	**Model 1**	**Model 2**
**Dependent variable**	**Firm size inequality at *t***	**Number of firms at *t***
Dependent variable at *t*_−1_	0.713^***^	1.00^***^
	(0.064)	(0.065)
Number of firms at *t*_−1_	−0.023^***^	
	(0.006)	
Firm size inequality at *t*_−1_		0.072
		(0.135)
Number of employees at *t*	0.041	0.148^***^
	(0.031)	(0.030)
Number of employees at *t*_−1_	−0.022	−0.083^***^
	(0.033)	(0.023)
Year dummies included (but not reported)	√	√
N industry-year obs./industries	706/63	706/63
Min./avg./max. obs. per industry (group)	2/11.2/12	2/11.2/12

*Firm size inequality at t is the dependent variable*.

*** *p < 0.001*.

*Conservative two-tailed tests for regressors and robust standard errors in parentheses*.

## Discussion

Panel data of Norwegian industries between 2001 and 2014 show that when they increase in the number of firms, firm size inequality in employees decreases. Decreasing firm size inequality implies that large firms become smaller in employees, and an increasing number of firms in an industry implies that more new firms are established than closed, i.e., ceasing to operate and going out of business. Thus, new firms chiefly recruit employees from large firms. Similarly, the data show that when industries decrease in the number of firms, firm size inequality in employees increases. Increasing firm size inequality implies that large firms become larger in employees, and a decreasing number of firms in an industry implies that more firms are closed than established. Thus, large firms chiefly recruit employees from firms that cease to operate. An implication of our findings is that large firms are crucial in recruiting employees to new firms and in recruiting employees from firms that cease to operate.

Our findings indicate that large firms are indeed crucial for entrepreneurship as they seem to prepare and “mold” talented people (Kacperczyk and Marx, 2016; Lee, 2021). Our findings also indicate that large firms are likely to recruit employees from firms that cease to operate. Despite working in a small firm having ceased to operate, we assume that entrepreneurial talent has been further molded because large firms would not otherwise recruit the employees. Taken together, our study shows that large firms and new firms, which inherently tend to be small and often cease to operate after a few years of operation (Aldrich, 1999; Wiklund et al., 2010), in a symbiotic relationship complement each other and are crucial for the development of entrepreneurial talent and employment in the private sector industry.

Taking an industry level, a limitation in our study is that we implicitly, but not explicitly, study the transfer of employees between firms. Therefore, we encourage future research to investigate firm- and employee-level mechanisms, e.g., age, gender, and education, that may act as genuine drivers or barriers concerning the transfer of employees from large firms to new firms, and to large firms from firms that cease to operate. Carrying out fixed effects regressions panel regressions, our study largely accounts for industry heterogeneity. Accordingly, we assume that our findings can be generalized beyond a limited context, but to further validate this assumption, future studies need to be carried out in other national contexts covering different time periods than we have studied.

## Data Availability Statement

The data analyzed in this study is subject to the following licenses/restrictions: Sharing of the data is restricted by Statistics Norway. Requests to access these datasets should be directed to jarle.aarstad@hvl.no.

## Author Contributions

JA conceptualized, developed, and wrote the paper. OK assisted in editing the text and in the execution of the econometric analyses. Both authors contributed to the article and approved the submitted version.

## Conflict of Interest

The authors declare that the research was conducted in the absence of any commercial or financial relationships that could be construed as a potential conflict of interest.

## Publisher's Note

All claims expressed in this article are solely those of the authors and do not necessarily represent those of their affiliated organizations, or those of the publisher, the editors and the reviewers. Any product that may be evaluated in this article, or claim that may be made by its manufacturer, is not guaranteed or endorsed by the publisher.
